# Gut Microbiome Analyses of Wild Migratory Freshwater Fish (*Megalobrama terminalis*) Through Geographic Isolation

**DOI:** 10.3389/fmicb.2022.858454

**Published:** 2022-04-08

**Authors:** Yaqiu Liu, Yuefei Li, Jie Li, Qiong Zhou, Xinhui Li

**Affiliations:** ^1^Pearl River Fisheries Research Institute, Chinese Academy of Fishery Sciences, Guangzhou, China; ^2^Key Laboratory of Freshwater Animal Breeding, Ministry of Agriculture and Rural Areas, College of Fisheries, Huazhong Agricultural University, Wuhan, China; ^3^Guangzhou Scientific Observing and Experimental Station of National Fisheries Resources and Environment, Guangzhou, China; ^4^Key Laboratory of Aquatic Animal Immune Technology of Guangdong Province, Chinese Academy of Fishery Sciences, Guangzhou, China

**Keywords:** gut microbiome, *Megalobrama terminalis*, geographic isolation, degradation enzymes, metabolism

## Abstract

Gut microbiome is considered as a critical role in host digestion and metabolic homeostasis. Nevertheless, the lack of knowledge concerning how the host-associated gut microbiome underpins the host metabolic capability and regulates digestive functions hinders the exploration of gut microbiome variation in diverse geographic population. In the present study, we selected the black Amur bream (*Megalobrama terminalis*) that inhabits southern China drainage with multiple geographic populations and relatively high digestive plasticity as a candidate to explore the potential effects of genetic variation and environmental discrepancy on fish gut microbiome. Here, high-throughput 16S rRNA gene sequencing was utilized to decipher the distinct composition and diversity of the entire gut microbiota in wild *M. terminalis* distributed throughout southern China. The results indicated that mainland (MY and XR) populations exhibited a higher alpha diversity than that of the Hainan Island (WS) population. Moreover, a clear taxon shift influenced by water temperature, salinity (SA), and gonadosomatic index (GSI) in the course of seasonal variation was observed in the gut bacterial community. Furthermore, geographic isolation and seasonal variation significantly impacted amino acid, lipid, and carbohydrate metabolism of the fish gut microbiome. Specifically, each geographic population that displayed its own unique regulation pattern of gut microbiome was recognized as a specific digestion strategy to enhance adaptive capability in the resident environment. Consequently, this discovery suggested that long-term geographic isolation leads to variant environmental factors and genotypes, which made a synergetic effect on the diversity of the gut microbiome in wild *M. terminalis*. In addition, the findings provide effective information for further exploring ecological fitness countermeasures in the fish population.

## Introduction

The vertebrate gut contains trillions of microorganisms collectively known as the gut microbiome (Budd et al., 2020). As aquatic vertebrates, the ecological interactions, digestion of materials, nutrient uptake, and energy acquisition of fish can be strongly influenced by their gut microbial community (Parris et al., 2016; Bird et al., 2019; Xu et al., 2019; Liu et al., 2021b). It has been confirmed that the importance of a gut microbiome to host health, fitness, and development with alterations of microbiome composition affects such properties as host nutrient acquisition, growth, reproduction, and even the whole population (Ghanbari et al., 2015; Parris et al., 2016). According to relative research, most fish hosts are essentially sterile during the earliest stages of development, and the gut microbiome is derived from the initial colonization by environmental microorganisms (Green and Bohannan, 2006; Goodrich et al., 2014; Stephens et al., 2016). Specifically, the gut microbial community in the environment can frequently be predicted by variations in environmental factors, such as salinity (SA) and temperature (Fortunato et al., 2012). Meanwhile, differentiation of the gut microbiome in the same species can also reflect variable exogenous environmental conditions (Fishelson et al., 1985; Su et al., 2020; Liu et al., 2021b). Some relevant research demonstrates that the diversity of the gut microbiome can also be partially interpreted by spatial and temporal variations among species, populations, and individuals (Lin et al., 2014; Bierlich et al., 2018; West et al., 2019). Fish hosts exhibit an autonomic selection of their gut microbiota, and the evolution of innate and adaptive immune systems is supposed to regulate the diversity and abundance of gut microbes. However, habitat environment, diet, and phylogeny are often interrelated, and their effects on the fish gut microbiome are prone to be complex and highly confounded. Moreover, intensive research of fish gut microbes has mainly focused on commercial or model species (Spor et al., 2011; Goodrich et al., 2014). How host geographic distribution affects the fish gut microbiome is still poorly understood. Thus, exploring the changes in the gut microbiome in wild hosts is considered an important way to help us to understand the ecological adaptability of the host's gut microbiota.

The black Amur bream (*Megalobrama terminalis*) is a migratory species that holds an important position in fishery production in southern China drainages. The river drainages located in southern China are complex and diverse. Sea-level fluctuations due to climate changes have been frequently documented to be a pivotal factor that shapes dynamic histories of drainages, particularly rivers near the sea, thereby influencing the geographic distribution of the *M. terminalis* population (Chen et al., 2020). On the basis of our previous research, *M. terminalis* exhibits three mtDNA genetic populations (the Pearl River, Moyang River, and Wanquan River population), each population presents a genetic structure related to the local geography (Chen et al., 2020). However, due to the continuous enhancement of human activities (e.g., water conservancy projects, waterway dredging, water pollution, and overfishing), the decline of wild *M. terminalis* populations has been reported in the Pearl River basin during this decade. Small population size, low genetic diversity, and fragmented distribution in the Moyang River and Wanquan River increase the risk of population extinction. In our latest research, *M. terminalis* was found to be an omnivorous fish with strong ecological adaptability (Liu et al., 2020). To supply sufficient energy for reproduction, *M. terminalis* could adjust its feeding habits, gut microbiome, and digestive activity to improve the efficiency of energy intake in the course of breeding migration (Xia et al., 2017; Liu et al., 2021b). Thus, *M. terminalis* is considered a good candidate for exploring the digestive plasticity of fish through habitats variation by geographic isolation.

According to related research, at the population level, local adaptation to the landscape may be reflected in the variable gut microbiome (Yuan et al., 2015; Trosvik et al., 2018), whereas the microbiome can be impacted by many endogenous and exogenous factors, such as host gender, phylogeny, habitat, and diet (Fishelson et al., 1985; Ley et al., 2008; Scott et al., 2013; Bergmann et al., 2015; Li et al., 2016; Aivelo and Norberg, 2018). To the best of our knowledge, most previous research on *M. terminalis* has focused on larval resources, feeding habits, ecological investigation of spawning grounds, and gonad development (Tan et al., 2009; Wang et al., 2010; Li et al., 2014; Xia et al., 2017; Liu et al., 2019). Nevertheless, the overall effect on the variation of the gut microbiome in the populations of *M. terminalis* through geographic isolation is poorly understood. To fill this knowledge gap, we attempted to investigate the diversity of the gut microbiome and their degradation enzyme activity in three wild geographic populations of *M. terminalis* in the dry (non-reproduction) and flood (reproduction) seasons to gain insights into the altering environmental conditions that drive the effect on the diversity of the gut bacterial community, which is of great importance for the research of its ecology and conservation biology. In parallel, illustrating the fine-scale variation in gut bacterial community of multiple *M. terminalis* populations can provide useful data for further exploration of host-associated microbiomes in the wild fish populations.

## Materials and Methods

### Sample Collection

**Figure 1:**
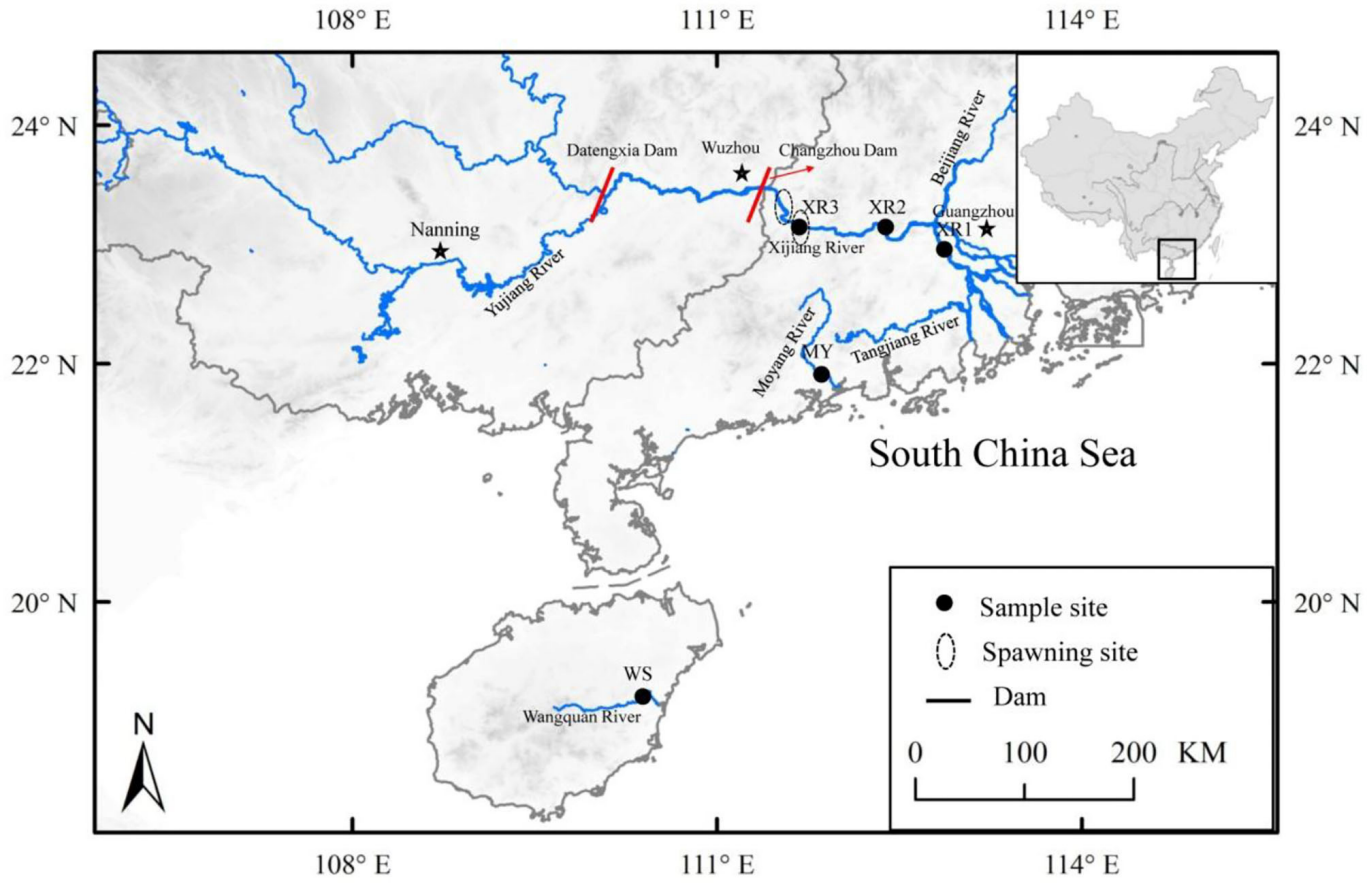
Locations of the sample sites for study areas, including three geographic populations of *Megalobrama terminalis*, distributed in South China. Black circles were expressed as the spawning grounds of *M. terminalis* in Pearl River.

In the present study, three geographic populations of the black Amur bream were sampled from six different sites in the Pearl River, Moyang River, and Wanquan River during the flood (July 2019) and dry (January 2020) seasons (Figure 1). A total of 200 wild *M. terminalis* specimens were collected. Water temperatures, SA, dissolved oxygen (DO), and pH of sampling sites were measured with an HQ30 instrument (Hach Company, Loveland, CO, USA). The sampling variables of time of collection, location, water temperature, SA, pH, and DO are listed in Table 1. Body standard length (SL, to the nearest 1 mm), body weight (W_t_, to the nearest 1 g), eviscerated weight (EW, to the nearest 1 g), gonad and liver weights (GW and LW, respectively, to the nearest 0.01 g) were measured. The gonadosomatic index (GSI = 100 × GW/EW), hepatosomatic index (HSI = 100 × LW/EW), and fatness (K = 100 × W/L^3^) were calculated. To evaluate the fish gut microbiome, 36 individuals (flood season *n* = 18; dry season *n* = 18) were randomly selected for sequencing from three populations: the Wanquan River (WS), Moyang River (MY), and Pearl River (XR) populations. All selected fish were anesthetized with an overdose of MS 222 (3-aminobenzoic acid ethyl ester methane sulfonate, Sigma, Germany) and stunned and quickly decapitated. The exterior surfaces of the fish body and instruments were wiped with 75% ethanol to prevent contamination from the skin surface, and instruments were sterilized before dissection. Approximately 0.4 g of gut contents were extracted for DNA extraction, and 0.2 g of gut contents were separated for use in enzymatic analysis; all contents samples were quickly put in liquid nitrogen and then transferred to an ultra-low temperature freezer and stored at −80°C until use.

**Table 1:** Basic environmental information, biological information of the different populations of *M. terminalis*.

		**WS**		**MY**		**XR**	
	**Season**	**Flood**	**Dry**	**Flood**	**Dry**	**Flood**	**Dry**
Environmental information	Sample period	July 2–9	January 4–9	July 10–17	January 10–15	July 17–July 27	January 16–26
	Temperature (°C)	30.1 ± 0.2^b^^*^	24.1 ± 0.3^b^	29.1 ± 0.4^a^^*^	19.5 ± 0.3^a^	28.7 ± 0.3^a^^*^	18.9 ± 0.4^a^
	Salinity (‰)	0.03 ± 0.01	0.09 ± 0.02^b^	0.01 ± 0.02	0.04 ± 0.01^ab^	0.01 ± 0.01	0.01 ± 0.00^a^
	pH	7.7 ± 0.2	8.1 ± 0.2	7.9 ± 0.2	8.2 ± 0.2	7.8 ± 0.2	8.2 ± 0.2
	DO (mg/L)	6.8 ± 0.2	6.4 ± 0.3	6.7 ± 0.2	6.3 ± 0.1	7.0 ± 0.2	6.6 ± 0.2
Biological information	*n*	30	30	30	30	30	30
	SL ± SD	232 ± 17.6^a^	222 ± 14.3^a^	254 ± 20.7^ab^	246 ± 22.4^ab^	272 ± 27.3^b^	261 ± 19.4^b^
	W_t_ ± SD	292 ± 19.4^a^^*^	177 ± 21.2^a^	427 ± 33.1^b^^*^	302 ± 25.7^b^	525 ± 30.1^c^^*^	406 ± 22.3^c^
	GSI (%)	4.9 ± 0.7^a^^*^	0.9 ± 0.3	8.3 ± 1.3^b^^*^	0.7 ± 0.1	10.8 ± 2.2^b^^*^	1.1 ± 0.4
	HSI (%)	1.0 ± 0.11^a^	1.3 ± 0.15^a^	1.8 ± 0.22^b^	2.4 ± 0.12^b^^*^	0.9 ± 0.10^a^	2.3 ± 0.11^b^^*^
	K	2.1 ± 0.13^a^^*^	1.6 ± 0.13^a^	2.4 ± 0.22^ab^^*^	1.9 ± 0.21^ab^	2.5 ± 0.24^b^^*^	2.0 ± 0.17^b^
	Sex mature ratio (%)	84.7	0	89.6	0	95.4	0

*Different superscript letters indicate significant differences in different populations, p < 0.05*.

*DO, dissolved oxygen; GSI, gonadosomatic index; HSI, hepatosomatic index; K, fatness; pH, pondus hydrogenii; SL, standard length; W_t_, body weight*.

* *Means significant difference between flood and dry seasons, p < 0.05*.

### DNA Extraction and Amplification

Approximately 0.2 g of each sample was extracted using a QIAamp DNA Stool Mini Kit (Qiagen, Valencia, USA). All DNA extracts were stored at −80°C until being tested. The quality and integrity of each DNA concentration and purity were monitored on 1% agarose gels. The V4 hypervariable region of 16S rRNA genes was used in the present study. PCR was performed using the specific primer (515F−806R) that contained a barcode. Total DNA from the gut of different groups of *M. terminalis* was sent to Novogene Bioinformatics Technology, Co., Ltd., Beijing, China for further sequencing analysis.

### High Throughput Sequencing Analysis

Sequencing libraries were created using the Ion Plus Fragment Library Kit 48 rxns (Thermo Scientific, USA) according to the manufacturer's instructions. The library quality was assessed on a Qubit@ 2.0 Fluorometer (Thermo Scientific, USA). After that, the library was sequenced, and single-end reads were generated. Quality filtering of the raw reads was performed under specific filtering conditions to obtain high-quality clean reads according to the Cutadapt quality-controlled process (Martin, 2011). The tags were compared with the reference database using the UCHIME algorithm to detect chimera sequences (Edgar et al., 2011; Haas et al., 2011). Sequence analysis was performed using UPARSE software (Edgar, 2013). Sequences with ≥97% similarity were assigned to the same operational taxonomic unit (OTU). The shared and unique OTUs of different groups were also represented by a Scale-Venn diagram. To compute the alpha diversity of fish gut microbiota, three metrics were calculated: Chao1, Ace, and Shannon indices were calculated with QIIME (version 1.7.0).

Permutation multivariate analysis of variance (PERMANOVA) was utilized to test the statistical significance of WS, MY, and XR populations (Anderson, 2001; Stat et al., 2013). MetaStat was used to compare species abundance between groups and to select species with significant differences (White et al., 2009). To explore the metabolic activity of the bacterial communities on the gut contents of different groups, Phylogenetic Investigation of Communities by Reconstruction of Unobserved States (PICRUSt) was utilized to analyze the Kyoto Encyclopedia of Genes and Genomes (KEGG) pathway at levels 2 and 3 (Langille et al., 2013).

### Enzyme Assays

The four specific activities (trypsin, amylase, cellulose, and lipase) were measured using corresponding enzyme assay kits from Nanjing Jiancheng Bioengineering Institute, P.R. China [A080-2 (trypsin assay kits), C016 (amylase assay kits), A138 (cellulase assay kits), and A054-2 (lipase assay kits)]. The soluble protein (mg/ml) contents were determined by the Bradford method using bovine serum albumin as the standard (0.563 g/L) (Bradford, 1976). All assays were performed on duplicate samples using an Infinite M200 Pro Tecan Sunrise (Tecan, Männedorf, Switzerland). UV-permeable Corning 96-well microplates (Corning Incorporated, Corning, USA) were used for all assays.

### Data Analysis

STATISTICA 6.0 (StatSoft, Inc., Tulsa, OK, USA) was used for statistical analysis of the data. The normality of the data and homogeneity of variance were assessed *via* the Kolmogorov-Smirnov test and Leven's test, respectively. In the WS, MY, and XR population, enzymatic activities were analyzed by one-way ANOVA. All data were expressed as means ± SD. A canonical correspondence analysis (CCA) was conducted to explain the relationship between the gut microbial compositions of different *M. terminalis* populations and their basic environmental and biological parameters and the correlation between gut microbial diversity and the degrading enzyme activity in different populations of the black Amur bream. Redundancy analysis (RDA) was conducted to explain the relationship between the KEGG orthologies (KOs) of the gut microbiome and the degrading enzyme activity in different populations of the black Amur bream. Here, we used the R implementation of the procedure (version 3.1.14).

## Results

### Gut Microbial Diversity and Composition

We obtained a total of 60,978 quality-filtered sequences from each sample. With a 97% sequence similarity cutoff value, the sequences were grouped into a total of 10,613 OTUs. OTU numbers, the alpha-diversity index (Shannon index), and richness indices (Chao1, Ace) were applied to evaluate the gut microbial complexity of the three populations (Table 2). It can be seen from data in Table 2 that the numbers of OTUs, Shannon index, and richness indices of the three geographic populations in the flood season are much higher than those of the dry season (*p* < 0.05). No matter whether the flood or dry season, the WS population exhibited the lowest alpha diversity and richness indices. Moreover, no significant differences in alpha diversity or richness were found between the MY and XR populations. In general, our results demonstrated that the gut microbiome in mainland populations had higher alpha diversity than that in the Hainan Island population. The Good's coverage of all the groups ranged from 98.87 to 99.53% (Table 2).

**Table 2:** Overview of operational taxonomic unit (OTU) numbers, alpha-diversity index, and richness estimator of the gut microbial community in the different geographic populations of *M. terminalis*.

		**WS**		**MF**		**XR**	
		**WSF**	**WSD**	**MYF**	**MYD**	**XRF**	**XRD**
Numbers of OTUs		2,059 ± 504^a^^*^	736 ± 208^a^	3,099 ± 574^b^^*^	1,198 ± 288^b^	2,934 ± 255^b^^*^	1,312 ± 233^b^
Alpha-Diversity index	Shannon	5.12 ± 0.36^a^^*^	4.06 ± 0.40^a^	7.85 ± 0.58^b^^*^	6.28 ± 0.34^b^	7.51 ± 0.35^b^^*^	6.83 ± 0.32^b^
Richness estimator	Chao1	2,102.63 ± 535.30^a^^*^	747.19 ± 237.55^a^	3,047.10 ± 611.21^ab^^*^	1,205.91 ± 286.51^b^	3,139.95 ± 263.71^b^^*^	1,328.22 ± 251.64^b^
	Ace	2,043.34 ± 495.07^a^^*^	735.89 ± 222.39^a^	3,075.18 ± 614.06^ab^^*^	1178.81 ± 286.49^b^	3164.42 ± 255.24^b^^*^	1301.74 ± 242.62^b^
Goods coverage %		98.87 ± 0.16	99.63 ± 0.13	98.65 ± 0.28	99.53 ± 0.18	98.61 ± 0.20	99.47 ± 0.14

*Different superscript letters indicate significant differences in different populations, p < 0.05*.

* *Means significant difference between flood and dry seasons in the same population, p < 0.05*.

**Figure 2:**
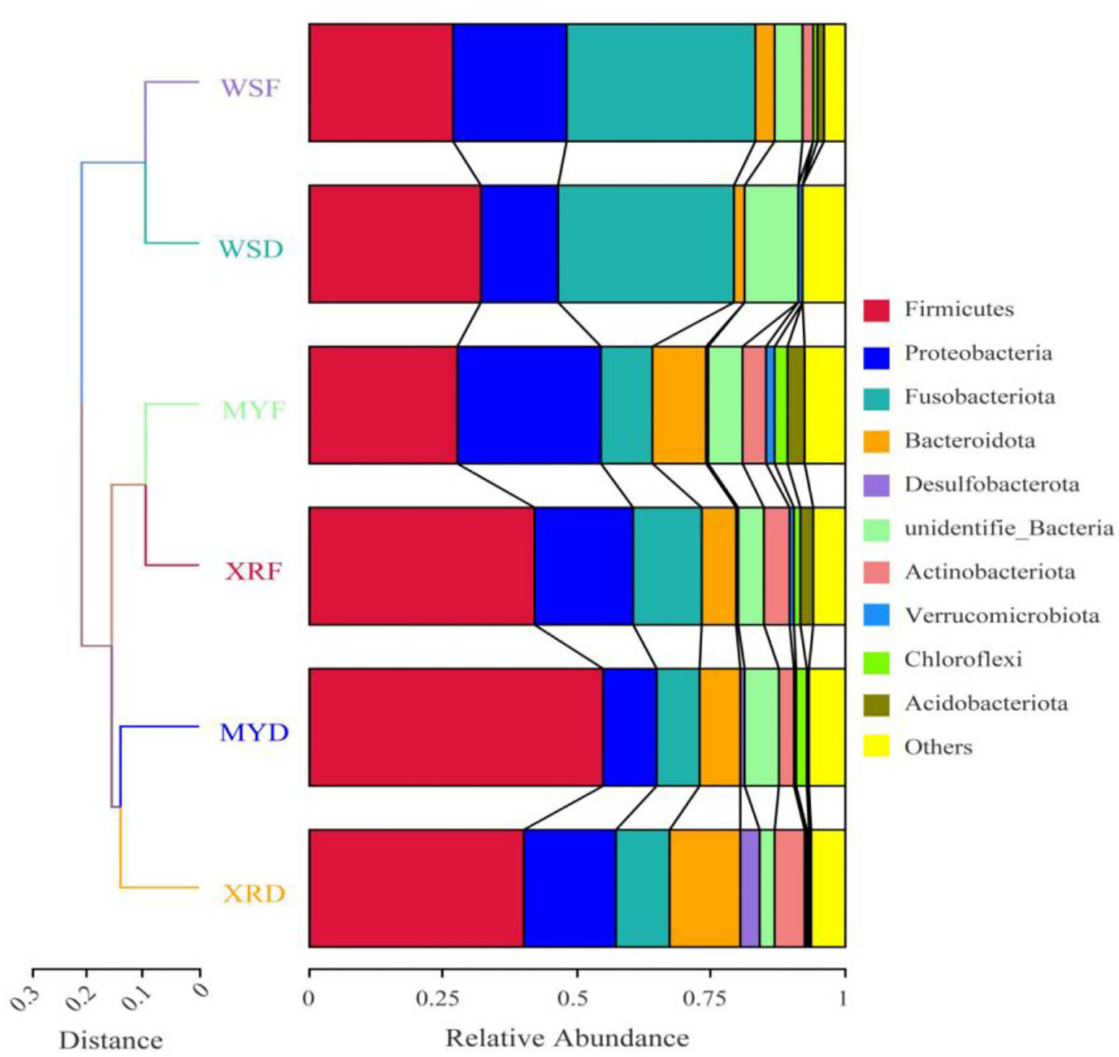
Comparison of a gut bacterial community in three geographic populations of *M. terminalis*. The unweighted pair group method with arithmetic mean (UPGMA) clustering tree of the gut bacterial community compositions of three populations in dry and flood season based on weighted UniFrac distance matrix is presented on the left side. Relative abundance of the top 10 phyla in three populations is shown on the right side.

To compare the similarity of gut bacterial community between different populations, the unweighted pair group method with arithmetic mean (UPGMA) clustering tree based on weighted UniFrac distances elaborated that MY and XR populations formed a cluster, while the WS population was a single cluster apart from the other populations. The bacteria were classified into 84 phyla, 161 classes, 351 orders, 549 families, and 1,260 genera (Figure 2). At the phylum level, Firmicutes were the most abundant in the XRF (42.05%), XRD (40.34%), and MYD (54.98%) groups, while the most abundant phylum of the WSF (35.40%) group was Fusobacteriota (Figure 2). The abundances of Firmicutes (27.71%) and Proteobacteria (27.11%) in the MYF group were similar, and the abundances of Firmicutes (32.21%) and Fusobacteriota (32.67%) in the WSD group were similar. PERMANOVA analysis revealed that the differences in groups were significant (*p* < 0.05, Table 3), except for MYF vs. XRF (*p* = 0.249).

**Table 3:** Permutation multivariate analysis of variance (PERMANOVA) analysis of the weighted UniFrac for three geographic populations of *M. terminalis*.

**Pair-Wise test**	***P*-value**
MY-WS-XR	<0.001^**^
WSD-WSF	0.004^**^
MYD-MYF	0.007^**^
XRD-XRF	<0.001^**^
MYF-WSF	0.004^**^
MYF-XRF	0.249
WSF-XRF	0.005^**^
WSD-XRD	0.013^*^
MYD-XRD	0.01^*^
MYD-WSD	0.029^*^

* *Means significant difference between two populations (p < 0.05)*.

** *Means very significant difference between two populations (p < 0.01)*.

### Spatial and Temporal Differences in Gut Bacterial Community

**Figure 3:**
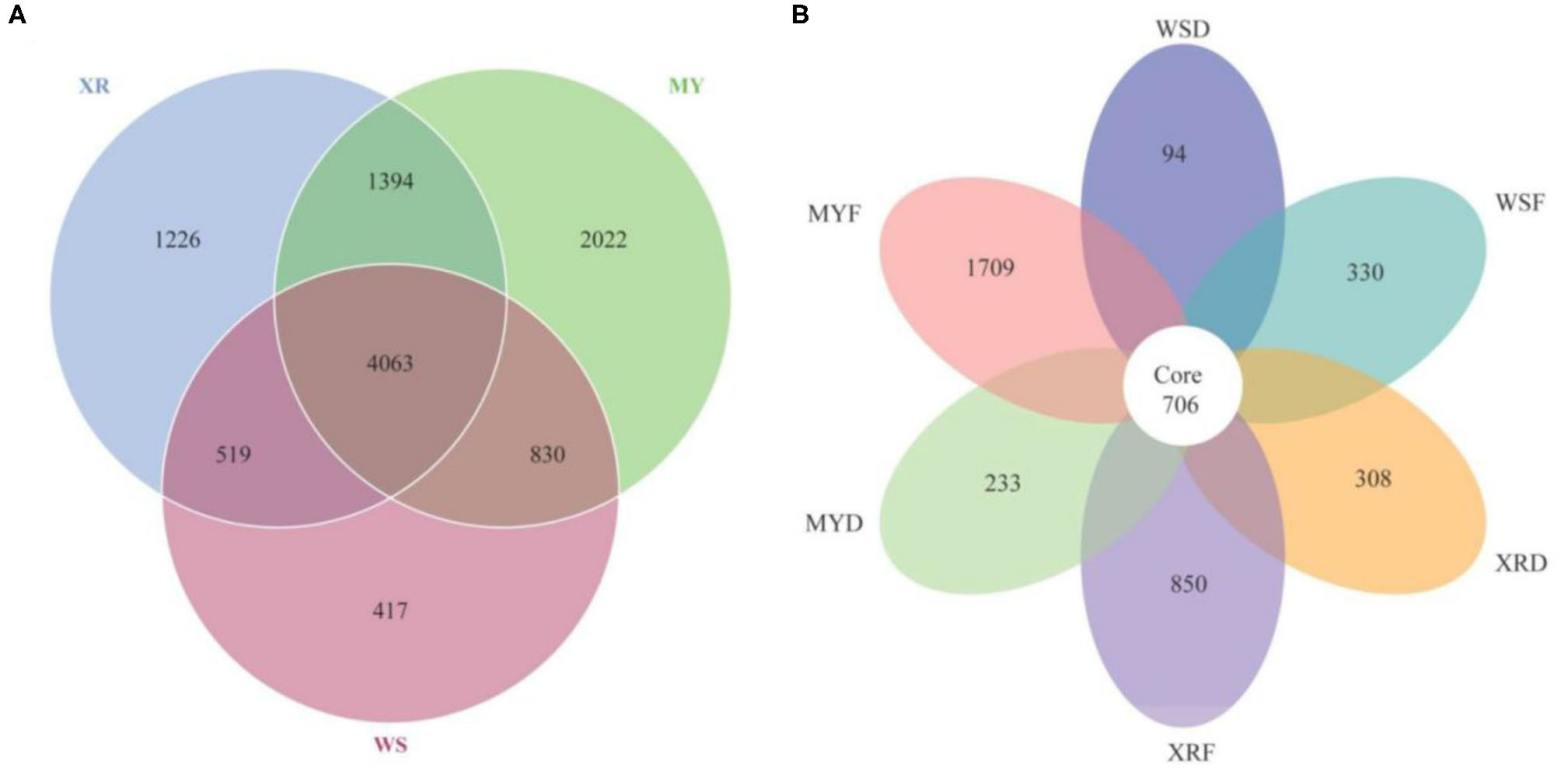
Venn diagram illustrates the shared and unique gut microbial species in the different geographic populations studied of *M. terminalis*. **(A)** The Venn diagram shows the number of shared and unique operational taxonomic units (OTUs) among three geographic populations. **(B)** The Venn diagram shows the number of shared and unique OTUs among three geographic populations in flood and dry seasons.

**Figure 4:**
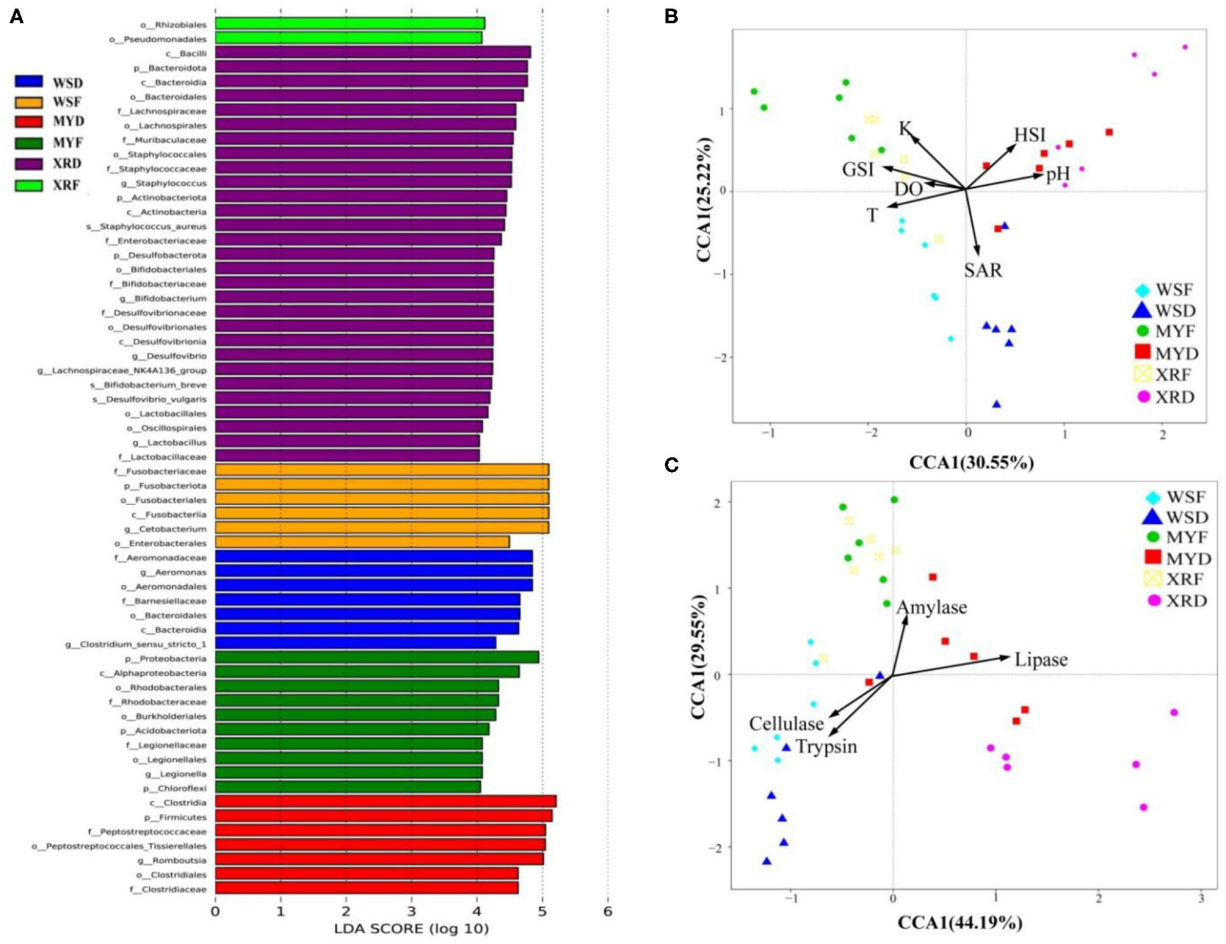
Spatial and temporal differences in gut bacterial community in three geographic populations of *M. terminalis*. **(A)** LEfSe identifies the most differentially abundant taxons among different geographic populations. For the species with significant differences in relative abundance for different groups and their effects, the linear discriminant analysis (LDA) scores (≥4) were listed and the higher score means bigger effects. **(B)** Canonical correspondence analysis (CCA) demonstrates the correlation between the gut microbial compositions of different populations and their basic environmental and biological parameters. **(C)** CCA indicates the correlation between the gut microbial compositions of different populations and their gut content enzyme activities. DO, dissolved oxygen; GSI, gonadosomatic index; HSI, hepatosomatic index; K, fatness; pH, pondus hydrogenii; SAR, salinity T, water temperature.

As can be seen in Figure 3, a Venn diagram is used to illustrate the shared and unique OTUs in different populations. A total of 5,829, 8,309, and 7,202 OTUs were observed in the WS, MY, and XR populations, respectively (Figure 3A). The WS, MY, and XR populations shared 4,063 OTUs (38.8%). The WS population had the fewest unique OTUs (4.0%), whereas the XR population exhibited the largest number of unique OTUs (19.3%). The number of common core OTUs present in the six groups was 706, and unique OTUs for each group varied from 94 to 1,709 (Figure 3B). For WSF groups, the genus *Cetobacterium* could be considered as a microbiological marker to differentiate WSF groups from the other groups. *Aeromonas* and *Clostridium* in the WSD group presented higher linear discriminant analysis (LDA) scores than those in the other groups (Figure 4A). On account of groups cultured in different seasons and habitats, the families, Legionellaceae and Rhodobacteraceae, were significantly different in the MYF group when compared to the other groups, whereas the families, Clostridiaceae and Peptostreptococcaceae, were significantly different in the MYD group. Samples from the XRF group, the relative abundance of the order Pseudomonadales was significantly higher. Compared with the other groups, the representative microbes in the XRD group were the genera *Staphylococcus, Bifidobacterium, Lactobacillus*, and *Desulfovibrio* (Figure 4A).

### Biological Parameters of Fish in Different Habitats

As seen in Table 1, water temperature in the flood season is much higher than that in the dry season in the habitats of three populations (*p* < 0.05). It is evident from the result that overall, there was a decreasing trend in SA with latitude change of habitat in the dry season (*p* < 0.05). The results of fish basic biological information revealed that SL, Wt, GSI, and K of WS, MY, XR populations were rising in turn. Meanwhile, it was exhibited that there were significant differences in SL, Wt, GSI, K, and sex mature ratio (SMR) in three populations between flood and dry seasons. In the flood season, HSI of MY was the highest among the three populations. The gut bacterial community composition in each group had a close relationship with their habitat environment and biological index (Figure 4B). The gut bacterial community composition of the MYF and XRF groups was more related to DO, fatness, and GSI. In contrast, the gut bacterial community composition of WSD and WSF was correlated with SA and water temperature. In brief, there were taxon shifts among the gut microbiota with both season and habitat variations.

### Degradation Enzymes Activity of Gut Contents

Gut content enzyme activities in the three geographic populations are shown in Supplementary Figure 1. As a whole, trypsin and cellulase activities of the WS population were higher than that of the XR and MY populations, whereas the amylase and lipase activities of WS population were lower than that of the XR and MY populations. Trypsin activity was declined and cellulase activity was increased from WSF to WSD groups (*p* < 0.05). Likewise, trypsin activity was decreased significantly while the amylase and lipase activities were clearly increased from MYF to MYD groups (*p* < 0.05). Lipase activity dropped off sharply, while amylase activity had a dramatic increase from the XRF to XRD groups. As shown in Figure 4C, the fish gut bacterial community has a close relationship with host metabolic enzymes. The gut microbiota composition of WSF and WSD groups was correlated with trypsin and cellulase activities but not with lipase or amylase activities. By contrast, the gut microbial compositions of the MYF and XRF groups were more related to amylase activities. MYD and XRD groups were more closely related to lipase activities.

### Function Analysis of Core Bacterial Community

**Figure 5:**
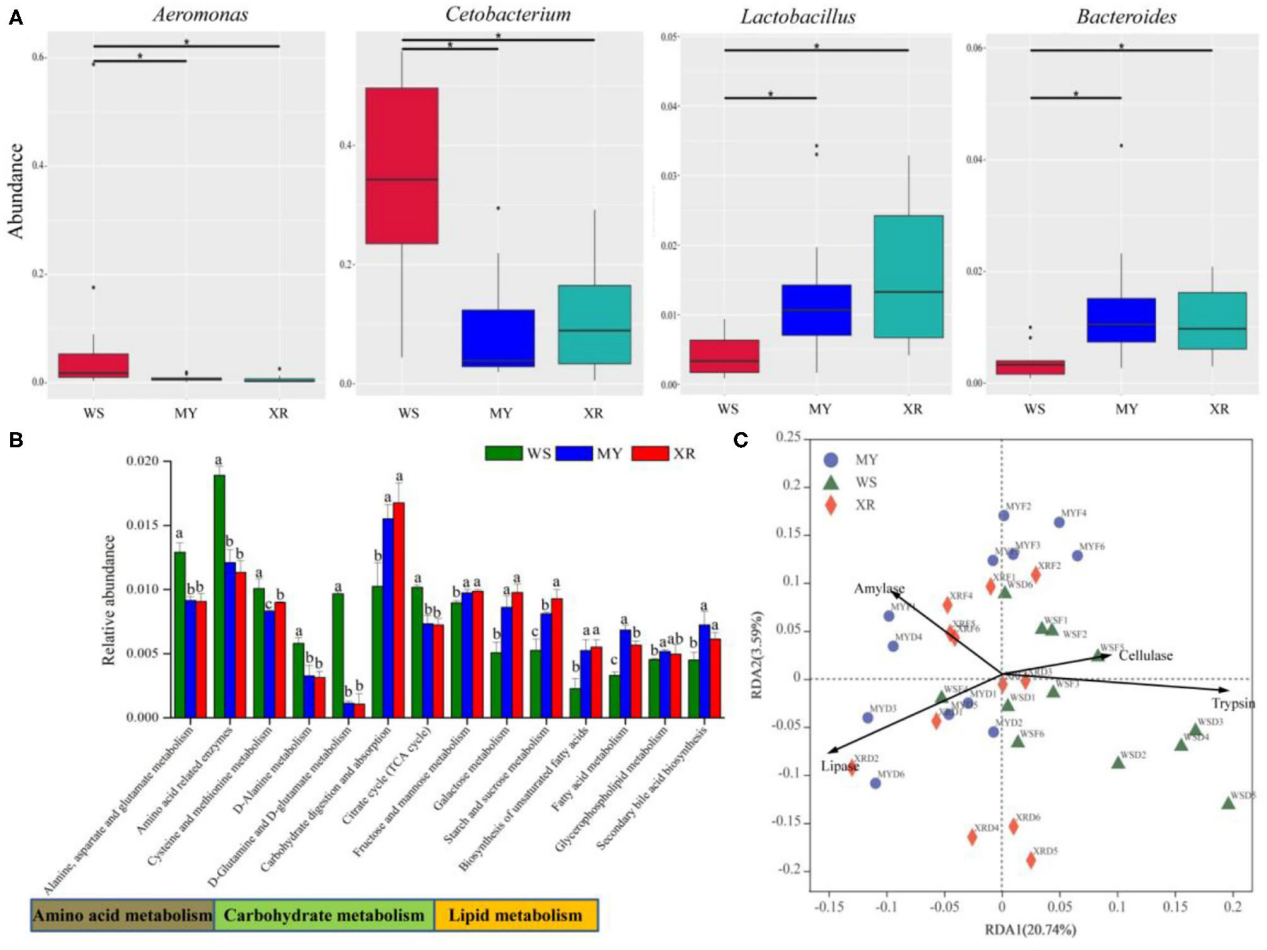
Functional analysis of gut core microbiome in three geographic populations of *M. terminalis*. **(A)** MetaStat analysis of gut core microbiome relative abundance of three populations in genera level. “*” means a significant difference between the two groups (*p* < 0.05). **(B)** The Kyoto Encyclopedia of Genes and Genomes (KEGG) categories are derived from the 16S rRNA sequences of the fish gut microbiome by Phylogenetic Investigation of Communities by Reconstruction of Unobserved States (PICRUSt). Comparison of the relative abundance of the selected KOs in three geographic populations of *M. terminalis*. Different superscript letters indicate significant differences in three geographic populations in gene categories at level 3, *p* < 0.05. **(C)** Redundancy analysis (RDA) illustrates the correlation between the dissimilarity of the functional profiles (KEGG level 3) of the gut microbiome in different populations and their gut content enzyme activities.

MetaStat was utilized to analyze the relative abundance among the three disparate populations at the genus level. From above Figure 5A, the results indicate that abundances of the genera *Aeromonas* and *Cetobacterium* in the WS population are significantly higher than that in the MY and XR populations (*p* < 0.05), while the genera *Lactobacillus* and *Bacteroides* are lower. According to Figure 5B, the relative abundances of 16 genes for carbohydrate metabolism, amino acid metabolism, other amino acids metabolism, lipid metabolism, and the endocrine system show statistically significant differences (*p* < 0.05; Figure 5B). Geographic isolation had significant effects on amino acid, lipid, and carbohydrate metabolism. Functional microbiota of the WS population related to amino acid metabolism was greater than in the MY and XR populations, while the relative abundances for lipid metabolism and carbohydrate metabolism in the XR population were greater than those in the WS population. RDA based on KOs predicted by PICRUSt and activity revealed that the functional gene of fish gut microbiomes had a close relationship with host degradation enzymes. The gut microbiota KOs of the WS population were correlated with trypsin and cellulase activities. By contrast, the gut microbial compositions of the MY and XR populations were more related to amylase and lipase activities (Figure 5C).

## Discussion

In the current study, we evaluated the gut microbiota and their degradation enzyme activities in three wild geographic populations of *M. terminalis* in the flood and dry seasons. Our results indicated that the XR population had higher alpha diversity than that of the WS population. Moreover, the UPGMA clustering revealed that the MY population was similar to the XR population that was aligned with a genetic divergence of three black Amur bream populations (Chen et al., 2020). Relevant research elaborated a positive relationship between gut microbiome distance and host genetic distance in three-spined stickleback (Steury et al., 2019). It seems likely that a substantial portion of gut microbiota variation is controlled by the host genotype (Smith et al., 2015). When seasonality and habitat environment factor in the mainland and Hainan Island populations was considered, a higher bacterial diversity was present in the flood season than that in three wild populations in the dry season. Converting in environmental factors by seasonal variation is regarded as one of the factors that can shift the gut microbiota (Dulski et al., 2020). Recent studies reveal that the gut microbial community can be altered by dramatic variation of habitat environment (Sullam et al., 2012; Dulski et al., 2020; Kim et al., 2021). In the present research, water temperature expressed a remarkable rise in the flood season (Table 1), which seems to be a critical factor for the shift of gut microbiota. Related research assumes that seasonal variation make effects on fish gut microbial composition by changing temperature (Nayak, 2010; Hatje et al., 2014). The result of PERMANOVA analysis clarified that the relatively geographic location and habitat environment of two populations might indirectly reflect their variant in the gut microbiome (Table 3). The result is consistent with a relevant study that fish gut bacterial community could be shaped by geographic determination and habitat environment (Sullam et al., 2012).

For the core gut microbiome at the phylum level, a relative abundance of Fusobacteriota in the WS population was significantly higher than that of the MY and XR populations, whereas the relative abundance of Bacteroidetes was lower. In grass carp, Fusobacteriota was considered as one of the most dominant flora (Wu et al., 2012). Meanwhile, Bacteroidetes is responsible for the metabolism of steroids, polysaccharides, and bile acids, helping the fish host in the absorption of polysaccharides and synthesis of proteins (Bäckhed et al., 2005; Xu et al., 2019). MetaStat results reflected that the relative abundance of *Aeromonas* and *Cetobacterium* in the WS population expressed significantly higher than that of the MY and XR populations, while the relative abundances of *Lactobacillus* and *Bacteroides* were lower than that of the WS population (Figure 5A). In the herbivorous fish *Ctenopharyngodon idella, Aeromonas* was thought to be pivotal cellulase-producing bacteria in the relevant study (Jiang et al., 2011). On the basis of related research, the carnivorous fish species are enriched with *Cetobacterium*, which has a close relationship with protein digestion (Liu et al., 2016). As vital gut microflora, *Lactobacillus* and *Bacteroides* contribute to fish host glucose and lipid metabolism (Samuel et al., 2008; Bjursell et al., 2011; Falcinelli et al., 2015). Furthermore, *Lactobacillus* isolated from the intestines of some animal can effectively degrade polyunsaturated fatty acids (Druart et al., 2014). In addition, PICRUSt functional analysis of the gut microbiome exhibited greater amino acid metabolism and lower lipid and carbohydrate metabolism in the WS population when compared with the MY and XR populations (Figure 5B). It is evident from the functional analysis result that overall, the gut microbiota of digestive functions in the three populations of *M. terminalis* presented differentiation by geographic isolation. A recent study clarifies that the gut microbiota is closely associated with host physiological metabolism, nutrient utilization, nutritional status, and health (Wu et al., 2021).

Considering the effects of geographic divergence and seasonal variation on gut microbiome composition and degradation enzymes of the black Amur bream, we investigated four key degradation enzyme activities that had a close relationship with the gut microbiome (Figure 4C). The evidence points to the likelihood that food digestion depends on the aid of gut symbiotic microorganisms to degrade macromolecules of nutrients in food and supply requisite energy to the fish. A further result indicated that there was a close relationship between four host degradation enzyme activities and functional profiles of the gut microbiome in three wild populations (Figure 5C). This work represents a preliminary attempt to explore a variation pattern of the gut microbiome and degradation enzymes in the course of seasonal changes in three wild populations. Seasonal variation not only leads to habitat environmental changes but also leads to fish sexual maturity levels. Our previous research work elaborated that the breeding peak of *M. terminalis* was from late June to early July (Liu et al., 2021a). Meanwhile, *M. terminalis* was found as a one-time spawning type of fish, with ovary development is partially synchronous (Liu et al., 2019). In the flood and the breeding seasons, a high abundance of *Cetobacterium* signified that the black Amur bream digest foods with higher nutrition levels to supply energy demand for spawning migration. In the dry season, the WS population transformed the core genus *Cetobacterium* to *Aeromonas* and *Clostridium*, marking enhancement of cellulose degradation capacity (Mcdonald et al., 2015). The transformation of core bacteria reflected that the gut microbiota of *M. terminalis* was altered to consolidate the cellulose metabolism to compensate for food shortages. However, the XR population displayed an obviously different transition mode of the core gut microbial community through seasonal changes. High relative abundance of the order Pseudomonadales in the XRF group indicated a heightened ability to metabolize fat (Lésel et al., 1989). A previous study demonstrates that the XR population migrates nearly 250 km upstream to the spawning grounds in the Pearl River (Liu et al., 2021a). Owing to the energy demand of the development of ovaries, the gut microbiome of *M. terminalis* might be regulated to enhance lipid metabolism. It is possible that *M. terminalis* might regulate the composition of the gut microbiome to improve the efficiency of nutrient metabolism to furnish enough energy for spawning migration. By analyzing the degrading enzyme of the MY population's gut content, it appears from the evidence that there were similarities and differences between the MY population and the other populations in their digestion strategies. In brief, this seems to imply that there is a diverse digestive strategy for *M. terminalis* survival and reproduction in three populations, respectively.

In conclusion, gut bacterial communities are assumed of great importance in fish digestion physiology and metabolic homeostasis. Multitudinous factors, such as driving environmental factors, diet, and genotype, synergistically impact on the fish gut microbiome (Bolnick et al., 2014; Liu et al., 2016; Escalas et al., 2021). In comparisons of gut microbiome composition in three geographic populations of *M. terminalis*, we found that each independent population shaped its own unique variation patterns of gut microbiome through altering environmental factors. This variation pattern of the gut microbiome was recognized as a specific digestion strategy in each geographic population. Due to geographic isolation, genetic and environmental differentiation jointly formed a relatively independent gut microbiome in different populations of *M. terminalis*. It might be reasonable to suppose that habitat environment was one of the primary factors for shaping the gut bacterial community, then disparate genotype further reinforced these differences in the gut microbiome, deeply influenced the fish's own specific digestive strategies for survival and breeding. Nevertheless, how the gut microbiota effects host food sources, food types, and nutritional content is still unclear. Therefore, the ecology and physiology of the fish gut microbiota should be considered in a future study.

## Data Availability Statement

The datasets presented in this study can be found in online repositories. The names of the repository/repositories and accession number(s) can be found in the article/Supplementary Material.

## Ethics Statement

The methods involving animals in this study were conducted in accordance with the Laboratory Animal Management Principles of China. All experimental protocols were approved by the Ethics Committee of the Pearl River Fisheries Research Institute, Chinese Academy of Fishery Sciences.

## Author Contributions

YLiu: conceptualization, data curation, and writing—original draft. XL: funding acquisition. JL: funding acquisition and supervision. YLi: formal analysis and writing—review and editing. QZ: formal analysis. All authors contributed to the article and approved the submitted version.

## Funding

This study was funded by Guangdong Basic and Applied Basic Research Foundation (Grant No. 2019B1515120064); the National Key R&D Program of China (Grant Nos. 2018YFD0900902 and 2018YFD0900903); Open Fund of Key Lab of Freshwater Biodiversity Conservation, Ministry of Agriculture and Rural Affairs of China (Grant No. LFBC1006); and Open Fund project of Fishery Resources and Environmental Science Experimental Station of The Upper-Middle Reaches of Yangtze River Ministry of Agriculture (Grant No. 0202020017).

## Conflict of Interest

The authors declare that the research was conducted in the absence of any commercial or financial relationships that could be construed as a potential conflict of interest. The reviewer CS declared a shared affiliation with the authors YLiu, YLi, JL, and XL to the handling editor at the time of review.

## Publisher's Note

All claims expressed in this article are solely those of the authors and do not necessarily represent those of their affiliated organizations, or those of the publisher, the editors and the reviewers. Any product that may be evaluated in this article, or claim that may be made by its manufacturer, is not guaranteed or endorsed by the publisher.
