# Supplementary material for: Gut Microbiome Analyses of Wild Migratory Freshwater Fish (Megalobrama terminalis) Through Geographic Isolation
Source: Front Microbiol. 2022 Apr 8;13:858454. doi: 10.3389/fmicb.2022.858454 (PMC9026196; doi:10.3389/fmicb.2022.858454)
Supplement: Supplementary file 1 [file Image_1.pdf]

SUPPLEMENT FIGURE

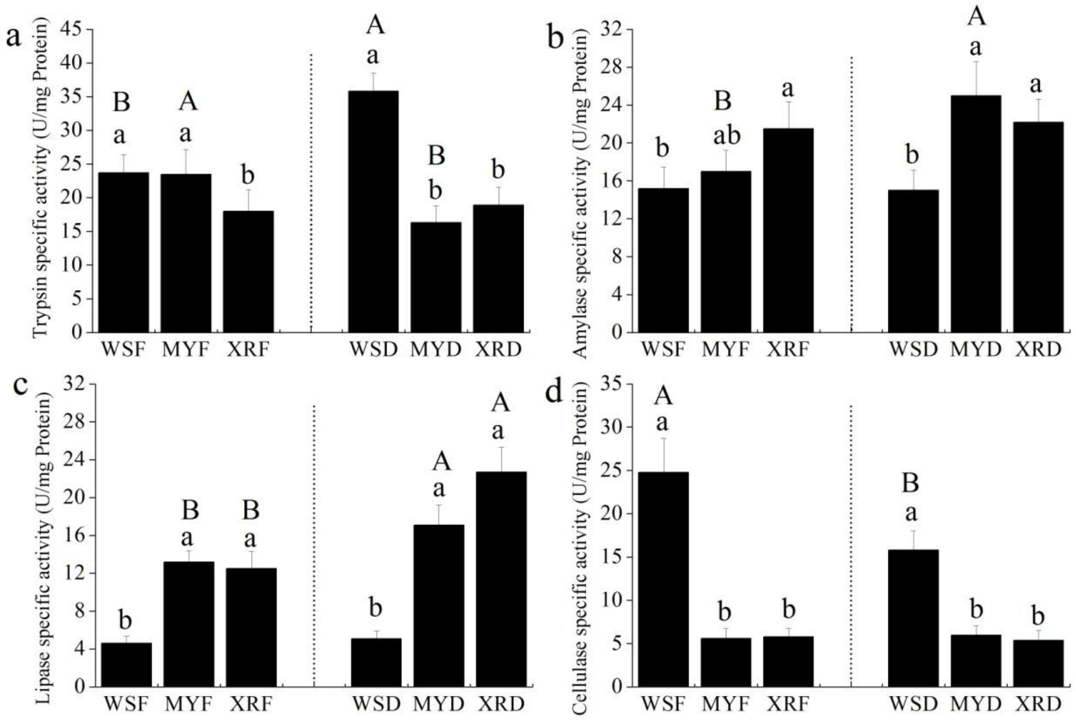

Fig. S1 Gut content enzymes activities in three geographic populations of *M. terminalis.*

(a) Trysin; (b) Amylase; (c) Lipase; (d) Cellulase

Note: Different lowercase letters indicate significant differences of enzymes specific activities in three populations in the same season (P < 0.05). Different capital letters means significant difference of enzyme specific activities of the same population in flood and dry season (P < 0.05).
